# Estimating the potential reduction in future sickness absence from optimizing group-level psychosocial work characteristics: a prospective, multicenter cohort study in German industrial settings

**DOI:** 10.1186/s12995-020-00284-x

**Published:** 2020-11-13

**Authors:** Joachim E. Fischer, Bernd Genser, Peter Nauroth, David Litaker, Daniel Mauss

**Affiliations:** 1 Mannheim Institute of Public Health, Mannheim Medical Faculty, University of Heidelberg, Ludolf-Krehl-Str. 7-11, 68167 Mannheim, Germany; 2 HealthVision GmbH, Hans-Bunte-Str. 8-10, 69123 Heidelberg, Germany

**Keywords:** Sickness absence, Predictability, Population attributable fraction, Psychosocial work characteristics, Prospective study, Health behavior, Cardiovascular risk, Multilevel cohort study

## Abstract

**Background:**

Absence from work due to sickness impairs organizational productivity and performance. Even in organizations with perfect work conditions, some inevitable baseline sickness absence exists amongst working populations. The excess sickness absence observed above this baseline rate has become the focus of traditional health promotion efforts, addressing preventable physical illness, health behavior and mental health at the personal level. However, a health and safety approach following the TOP-rule would consider work-group psychosocial work characteristics as a potential risk factor amenable to organizational measures. To date, there is a scarcity of studies relating psychosocial work characteristics to possible reduction of excess sickness-absence rates.

**Methods:**

We aimed to estimate the potentially avoidable excess fraction of absence attributable to work-group psychosocial characteristics. We considered work-group averaged perception of psychosocial work characteristics as a proxy to the methodologically elusive objective assessment of organizational characteristics. Participants were recruited from multiple sites of a German automotive manufacturer with individuals nested within work groups. We predicted 12-month follow-up work-group sickness absence rates using data from a baseline comprehensive health examination assessing work characteristics, health behavior, and biomedical risk factors. We considered the quartile of work-groups yielding favorable psychosocial work characteristics as a realistic existing benchmark. Using the population attributable fraction method we estimated the potentially amenable sickness absence from improving work-group psychosocial characteristics.

**Results:**

Data from 3992 eligible participants from 29 work groups were analyzed (39% participation rate, average age 41.4 years (SD = 10.3 years), 89.9% males and 49% manual workers.). Work-group absence rates at follow up varied from 2.1 to 8.9% (mean 5.1%, 11.7 missed days). A prediction model of seven psychosocial work characteristics at the work group level explained 70% of the variance of future absence rates. The estimated reduction from improving psychosocial work characteristics to the benchmark level amounted to 32% of all sickness absence, compared to a 31% reduction from eliminating health behavioral and medical risk factors to the benchmark target.

**Conclusions:**

Psychosocial characteristics at the work-group level account for a relevant proportion of all sickness absence. Health promotion interventions should therefore address psychosocial characteristics at the work group level.

**Supplementary Information:**

The online version contains supplementary material available at 10.1186/s12995-020-00284-x.

## Background

Maintaining a healthy and productive workforce is paramount for business organizations [1, 2]. Worker absence due to sickness has negative impacts on company productivity and performance [3] and places significant burden on social security and health care systems [4]. Due to the human nature however, largely “inevitable” diseases such as infections, accidents, cancer represent an unavoidable baseline sickness rate. The excess rate above this is deemed potentially preventable. To ameliorate excess sickness attributable to physical work conditions, organizations in industrialized nations have established a culture of health and safety. Safety measures are implemented to minimize sick leave directly associated with work [5].

During the past decades, it has been increasingly recognized that addressing health risks beyond direct work-related safety issues might further reduce employee’s sickness absence. Many companies for example now offer comprehensive vaccination programs including influenza immunization. Beyond vaccination, health promotion programs are intended to address modifiable risk factors for disease, such as nutrition, lack of physical activity, and smoking or alcohol consumption [6]. These efforts rest on the assumption that improved individual health behavior will reduce the likelihood of illness and thereby ultimately also positively affect sickness absence [7]. However, a recent rigorous cluster randomized controlled trial scrutinizing a sophisticated workplace wellness program failed to show changes in clinical measures of health, health care spending and utilization, or in employment outcomes after 18 months of follow-up [8].

Medically certified sickness absence from mental illness has almost doubled in Germany during the past 15 years [9]. Hence, several workplace wellness programs now additionally aim to strengthen individual’s mental resilience for example by means of employee assistance programs addressing the problem at the personal prevention level. Linking individual-level self-reported data to national registries, Danish researchers estimated that seven work-related psychosocial factors account for 29% of all sickness absence [10–12]. The Whitehall study implicated perceived low control combined with high demands as well as perceived deterioration in psychosocial work environment as risk factor for subsequent sickness absence [13–15]. As these studies followed individuals they could methodologically not disentangle self-reported individual perception from work group level exposure [16]. Thus, it remains elusive whether the observed effects arise from personality – work environment interactions or whether objectively assessed psychosocial work characteristics per se might affect sickness absence rates.

To date, only few studies applied a multilevel approach to elucidate the link between characteristics of work group psychosocial environment and work group absence [17]. Relying on self-reported data, but not on independently ascertained absence records, researchers related a specific psychosocial work characteristic, perceived collective autonomy, to sickness absence. Yet, there remains a research gap simultaneously considering a broader range of psychosocial work characteristics, health behavior and medical findings to explain the stark differences in sickness absence rates observed between work-groups of the same company [18].

From a health and safety perspective it is highly desirable to enumerate the proportion of excess sickness absence attributable to psychosocial work characteristics at the work-group level. Should this proportion be relevant, occupational health and safety policies (TOP-rule) mandate to first focus on technical and organizational changes and then offering personal protection such as individual mental resilience trainings and employee assistance programs. To elucidate the proportion of excess sickness absence attributable to psychosocial characteristics at the work-group level, we therefore prospectively studied an organization where structural information (individuals nested within work groups) as well as the relevant outcome (objectively recorded work group overall sickness absence) was available.

## Methods

### Aims and objective

The objective of this study, was to assess a) the amount of variation between work-groups in future sickness absence rates explained by work-group level psychosocial work characteristics in comparison to other health related information expressed as explained variance of future sickness absence rates by concurrently available information and b) to estimate the excess sickness absence rate observed in work-groups with impaired psychosocial work characteristics as compared to the most favorable quartile of work-groups, operationalized using the population attributable fraction concept. The population attributable risk (by some authors referred to as etiological fraction) offers an estimate for the proportion of a given risk (i.e. death, disease, sickness absence) that might theoretically be removed if all exposed subjects had the lowest level of exposure (i.e. work groups with favorable psychosocial work environment) [11, 19–21].

### Conceptual model and operationalization

The underlying conceptual model derived from the Whitehall II studies posits that multiple, albeit often correlated compositional factors (i.e. age, manual vs. non-manual work), health behaviors, medical outcomes and psychosocial risk factors or resources are prospectively related to sickness absence rates [14, 15]. Rather than individual sickness absence data from a potentially biased sample, the most relevant work-group level outcome is the objectively recorded sickness absence rate including every employee. Likewise, the ideal exposure measure would be an objective external measurement of psychosocial work characteristics. Yet, constructs such as perceived appreciation are almost impossible to measure externally. The next best option to obtain a measure is to ask every employee and to average these ratings, like school grades from a class. Unfortunately, participation rates vary between work-groups, introducing unknown selection or recruitment biases. An option to estimate the size of this bias is to randomly re-sample from the existing participants and to observe the obtained variation. Then, appropriate multivariable regression models provide a useful estimation of the true underlying relationship [22].

### Study setting and study population

The data for this prospective cohort study [23] were at seven work sites operated by a large German automotive manufacturer over a three-year period. As part of the program roll-out in 2014 and to maximize the potential generalizability of study results, the company’s human resource management team at each site selected work groups involved in production, engineering, development and administration and representing the range of work group sickness absence rates observed at the time of planning the program (see discussion section for consideration of possible selection bias).

All permanent employees aged 18 to 65 in the selected work-groups (size ranging from 25 to 1480) were eligible. For this study we included work-groups where at least 10 participants had specifically consented to scientific evaluation of their anonymized data (average consent rate 67%). A work-group was defined as the organizational unit in the organigram of the company that has one clearly identified superior and clearly identified organizational purpose. We excluded work-groups affected by a company-wide reorganization in 2015. These criteria resulted in 3992 participants from 29 work-groups from the original 5444 employees partaking in the comprehensive health evaluation in 2014. To address the issue of unequal work-group sizes in our analysis, we used a repeated bootstrap random sampling procedure as explained in detail below, which samples a larger proportion of participants from small work-groups (i.e. 70%) as compared to large work-groups (i.e. 5%)).

### Baseline measurements

Comprehensive evaluations at baseline, conducted between June 23rd and December 15th 2014, consisted of self-completed health questionnaires, detailed medical examinations performed by members of the company’s occupational health services and an assessment of psychosocial work characteristics. Participants recorded age, gender and main type of work (manual vs. non-manual). All of these variables or measures explained in detail below were considered as candidate predictors potentially related to future sickness absence.

Perceived health and health-related behaviors were assessed using the SF-12 for health-related quality of life (mental and physical summary scale) [24], the Copenhagen burnout inventory [25], daytime sleepiness [26], the IPAQ [27] for physical activity, the AUDIT-C [28] for alcohol consumption, and eight additional items assessing nutritional habits and current smoking status. The questionnaire further assessed self-reported regular intake of medications and physician approved medical conditions.

The detailed medical examination included body-mass-index, waist circumference, blood pressure, lipid levels, glycosylated hemoglobin (HbA1c), C-reactive protein, criteria for the metabolic syndrome based on the new IDF definition [29]. As no fasting glucose was obtained, we used a HbA1c level exceeding 5.7% (prediabetes) instead of the glucose > 100 mg/dl criterion. Cardiovascular risk was estimated using the Framingham-Algorithm [30]. To adjust for age effects, we calculated a “Framingham relative risk index” as the absolute predicted risk for an individual divided by the absolute predicted risk for a non-smoking, non-diabetic person of the same age and gender with other variables at the upper boundary of the most favorable quartile for all participants.

Psychosocial characteristics of the work environment were evaluated using a self-completed questionnaire based on the Copenhagen Psychosocial Questionnaire (COPSOQ V1, German version, that includes scales on quality of leadership, cognitive stress perception, quantitative and emotional demands, influence at work, predictability, job-satisfaction, possibilities for development, meaning of work, social support from colleagues and work life conflict [25]. Psychosocial work characteristics can either act as resource or as adverse factor. For example, recognition or supportive leadership are consistently viewed as a resource. For the context of this investigation, we defined either stressors (e.g. high demands) or lack of resources (e.g. low resources such as lack of supportive leadership) as potentially adverse work characteristics.

Work ability was assessed using the 22-item Workability Index (range: 7–49) [31] which also contains one item on self-reported sickness absence during the past 12 months. The latter was used to assess possible selection bias in sampling as described below. Questionnaires were either completed online or by use of a paper-pencil version. For each candidate variable where a work-group environmental exposure was conceptually conceivable (i.e. quality of leadership) we calculated the work-group average as the mean of the randomly selected participants (see below) and the individual perception as the difference of the work-group mean and the individual value.

With the exception of the SF-12, most of the used scales use some arbitrary enumeration directly derived from converting Likert-scale coding (e.g. 0–5) to total scores. To enhance comparability of scales using such different metrics, we report data in a transformed fashion that was employed to facilitate communication with management resembling the grading experienced by most managers during the final years in German high schools. There the best grade is 15 points and the population averages around 10 points and giving rise to a standard deviation of 2.2 points. This has been the standard reporting system in the Mannheim Industrial Cohort Study, with expected German working population average scores of 10.

The work ability index is widely used self-administered questionnaire capturing seven dimensions of health including present and expected future work-ability. Dimension scores add to a total ranging from 7 (unable to work) to 49 (excellent work ability). The work ability index and its short form predict future long-term sickness absence (area under the receiver operating characteristic curve = 0.82 for manual workers and 0.79 for non-manual workers [32].

### Outcomes

We obtained the work-group sickness absence from the official company records for 2014 and 2015. The averaged work-group sickness absence rate was determined from the proportion of total workdays missed during 2015 due to sickness for all members of each work-group (both participating individuals and non-participants). Total workdays in a year was assumed to be 220 after excluding holidays and the average number of days of vacation for employees throughout the company. For example, a sickness absence rate of 5% for a work-group of 100 employees implies that 1.100 of the 22.000 possible workdays in 2015 were lost due to sickness absence.

In Germany, any sickness absence longer than three days requires a physician’s medical certificate with an indication provided to the employer of the duration of the certified sickness leave. For up to six weeks of cumulative sickness absence per 12 months, the employer has to continue paying the salary. Thereafter, employees receive renumeration from the statutory health insurance. Thus, company records combining short-term non-medically certified absence and > 3 day medically certified absence are the most accurate source available, above any social security registry data. Due to the European General Data Protection Regulation no further detail on individual sickness absences was available from company records. Due to these restraints, the company required a minimum number of employees per work-group. Thus, in highly fragmented engineering departments with small work-groups, the study work-group operationalization did not represent the lowest organizational level.

### Statistical analysis

As explicated above, the outcome was measured sickness absence rates for all employees in 2015 at the work-group level, while candidate predictors were measured during the baseline assessment in 2014 at the individual level [33]. To address the possibility of participation selection bias, we compared the self-reported sickness absences from the baseline with the company records for sickness absence averaged for all employees from the included work groups [34].

**Fig. 1:**
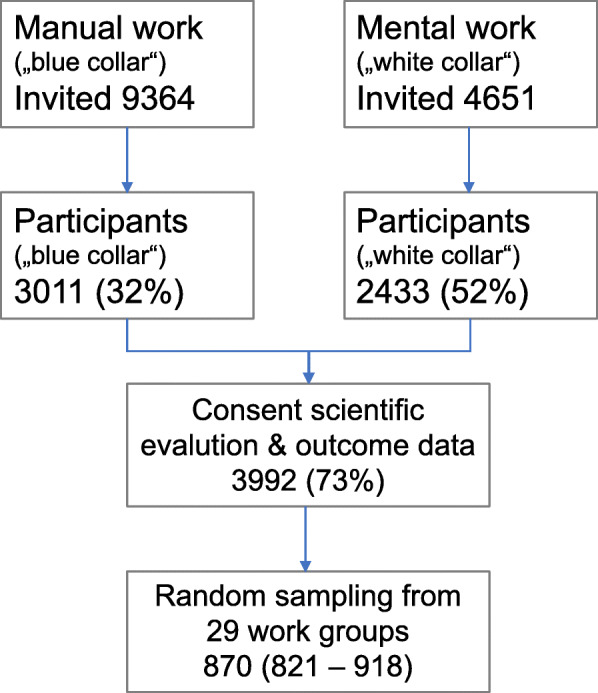
Recruitment of participants and analytical sample (repeated weighted random sampling)

Aggregating all data to the work group level would have obscured important information such as within work group heterogeneity (i.e. work groups with a differing distributions of characteristics).Thus, to account for these multilevel analytical structure issues, we employed a bootstrap procedure as recommended for complex data [35] in which individuals from each work-group are randomly selected to form samples for analysis. We used a sampling rate of 0.7 for small work-groups and 0.05 for the largest work-group. Random sampling was repeated for 200 cycles to allow the generation of empirical confidence intervals. This method resulted in an average total of 870 participants for the analytical sample (95% range 821–918). In all analyses we employed generalized linear models with a binominal logit link function. This acknowledge that the observed outcome rate (i.e. 5%) arises from multiple binary events (an employee being absent or present). We also conducted least-square linear regression analyses that essentially yielded similar results (data not shown) (Fig. 1).

First, we employed univariate analyses to establish the strength of the independent association of each candidate factor from psychosocial work characteristics, medical findings, self-reported health, health behaviors, compositional and contextual factors identified at baseline in 2014 with future sickness absence. For each candidate variable we explored linear and quadratic terms to account for possible non-linearity of the relationship. To facilitate interpretation of the results, we show univariate associations as effect sizes, expressed as explained variance of the prediction vs. observed data (R-square) [36].

In the multivariable analyses we explored combinations of candidate predictor variables for psychosocial work characteristics, medical information and health behavior, subjective health and compositional characteristics (age, gender, main type of work). For each model, the predicted sickness absence rate was compared to the observed absence rate using least square regression. Predictive accuracy was expressed as variance explained (adjusted R-square).

We expected substantial correlation across the medical and behavioral variables and amongst the psychosocial work characteristics. Therefore, we used backward elimination strategies to arrive at parsimonious models. Variables were eliminated until further reduction lead to an average decline in adjusted R-square over 200 bootstrap sampling cycles by more than 0.03. All analyses were repeated adjusting for age, gender and main type of work.

As the last analytical step, we estimated the excess sickness absence over the expected “unavoidable sickness absence” attributed to the set of predictors by the respective parsimonious models. For each of the models we determined the sickness absence rate for the quartile of work-groups with the most favorable prediction scores. Following the method originally suggested by Miettinen [19] we calculated the population attributable risk, also known as etiological fraction (PAF) [11, 21]. The PAF calculates the excess work-group sickness absence that would theoretically be removed if all work-groups had favorable psychosocial work-group characteristics. Here, we present a slightly different conceptualization, the excess absence expressed as proportion of unavoidable baseline sickness.

All analyses were carried out in STATA (StataCorp. 2017. *Stata Statistical Software: Release 15*. College Station, TX: StataCorp LLC.).

## Results

### Study population

The mean age of participants was 41.4 years (SD = 10.3 years), 89% were males and 49% were manual workers. Work-group composition ranged from almost entirely manual working environments to entire non-manual work-groups. Participation of employees across work-groups varied with an average rate of 39% (range 25–73%). Participant characteristics and grand means over work-groups are provided in Table 1. We observed substantial correlations across the medical and behavioral variables and amongst the psychosocial work characteristics (see supplement data).

**Table 1:** Baseline data of participants and work-groups. Group denotes group of predictors (c = compositional or work context, m = individual medical findings, hb = individual health behavior, psy = psychosocial work characteristics, wai = work ability). The left columns present the means and standard deviation over all participants, the right columns present the grand mean over the work-group means

		Participants (*N* = 3992)		Work-groups (*N* = 29)		
Parameter	Group	Mean	SD	Mean	SD	unit
Age	c	41.4	10.29	42.8	4.0	years
Manual work	c	49		58		percent
Gender (male)	c	89		82	22.0	percent
Body mass index	m	26.6	4.14	26.8	1.1	kg/m^2^
Diastolic blood pressure	m	82.2	9.15	82.4	3.4	mm Hg
Systolic blood pressure	m	129.1	13.84	129.2	4.7	mm Hg
Cholesterol	m	212.5	40.2	210.9	7.9	mg/dl
HDL-Cholesterol	m	50.8	11.6	50.4	3.2	mg/dl
LDL-to-HDL-Ratio	m	3.0	0.93	3.0	0.24	
Triglycerides	m	147.6	86.5	148.8	22.4	mg/dl
C-reactive protein	m	2.0	3.50	2.1	0.5	mg/l
γ-glutamyltransferase (GGT)	m	37.0	34.28	36.8	6.7	U/l
Glycosylated hemoglobin (HbA1c)	m	5.3	0.88	5.3	0.1	percent
Waist circumference	m	93.6	12.27	94.7	4.2	cm
Metabolic syndrome	m	1.4	1.16	1.4	0.3	criteria
Framingham score risk	m	7.9	7.38	8.1	2.2	percent
Framingham relative risk index	m	1.7	1.01	1.7	0.3	
AUDIT-C index	hb	10.2	2.98	10.3	0.8	points^a^
Physical activity index (IPAQ)	hb	10.4	3.83	10.6	1.2	points^a^
Number of medical conditions	hb	1.6	1.60	1.7	0.3	n
Smokers (self reported, percent)	hb	24.5		25.6	11.2	percent
Number of regular medications	hb	0.3	0.66	0.3	0.1	n
Nutritional index	hb	10.2	2.66	10.2	0.8	points^a^
Commitment to the workplace	psy	10.6	2.03	10.8	0.5	points^a^
Quality of leadership	psy	11.2	2.21	11.1	0.6	points^a^
Cognitive stress	psy	10.7	2.61	10.7	0.6	points^a^
Emotional demands	psy	10.6	1.74	10.6	0.5	points^a^
Physical demands	psy	10.8	2.26	10.8	1.0	points^a^
Quantitative demands	psy	9.3	2.15	9.2	0.8	points^a^
Influence at work	psy	10.2	2.51	10.3	0.8	points^a^
Predictability	psy	11.0	2.37	11.0	0.6	points^a^
Job satisfaction	psy	11.3	1.86	11.2	0.5	points^a^
Possibilities for development	psy	10.6	2.74	10.7	1.1	points^a^
Meaning of work	psy	11.7	2.15	11.9	0.5	points^a^
Social support from colleagues	psy	10.9	2.02	10.9	0.4	points^a^
Work life conflict	psy subh	10.7	2.29	10.7	0.7	points^a^
Burnout	subh	10.3	2.15	10.2	0.4	points^a^
SF-12 physical summary index	subh	9.9	2.80	9.7	0.8	points^a^
SF-12 mental summary index	subh	9.9	2.78	9.8	0.6	points^a^
Daytime sleepiness	subh	9.9	3.03	9.7	0.6	points^a^
Work ability	wai	40.7	5.85	40.5	1.4	points (7–49)

Legend: ^a^ The underlying scales have vastly differing metrices. To facilitate comparison across scales we standardized all scales to a working population mean of 10 with a standard deviation of 2.2 for a population of German industrial employees recruited to the Mannheim industrial cohort study between 2009 and 2013

### Absence rates

Sickness absence rates in the 2015 work year varied substantially across the 29 work-groups with a work-group mean rate of 5.1% (11.7 missed working days per year and employee), ranging from 2.1 to 8.9%. Work-group average sickness absence rate slightly increased during follow up compared to the 2014 baseline of 4.6% (10.6 missed days). Our assessment of possible participation selection bias showed that self-reported sickness absences from the baseline questionnaire average across individual participants amounted to 2.7%, while the company records for sickness absence averaged for all employees from the included work groups was 4.6%. The observed work-group absence rate correlated with the averaged self-reported sickness absence rate per work-group 2.7% (r = 0.49, *p* < 0.001). Forty percent of the participants reported no sickness absence at all.

### Prediction models

Table 2 presents the results from a univariate analysis relating candidate predictor variables to subsequent work-group absence rates at follow-up. Variables from the medical domain, health behavior (e.g., smoking) and psychosocial domain showed small to moderate effect sizes in predicting future sickness absence.

**Table 2:** Univariate associations of candidate predictors with sickness absence, explained variance

Parameter	Group	Sq^a^	R-square^b^
Age	c		0.09 (95%CI: 0.01–0.2)
Manual work (percent)	c		0.35 (95%CI: 0.32–0.39)
Gender (males, percent)	c		0.12 (95%CI: 0.02–0.24)
Body mass index	m		0.21 (95%CI: 0.09–0.35)
Diastolic blood pressure	m		0.08 (95%CI: 0.02–0.18)
Systolic blood pressure	m	X	0.17 (95%CI: 0.07–0.3)
Cholesterol	m	X	0.04 (95%CI: 0–0.1)
HDL-Cholesterol	m		0.42 (95%CI: 0.28–0.57)
LDL-Cholesterol	m	X	0.04 (95%CI: 0–0.13)
Triglycerides	m		0.27 (95%CI: 0.13–0.45)
C-reactive protein	m	X	0.19 (95%CI: 0.05–0.37)
γ-glutamyltransferase (GGT)	m	X	0.22 (95%CI: 0.07–0.39)
Glycosylated hemoglobin (HbA1c)	m	X	0.23 (95%CI: 0.05–0.4)
Waist circumference	m		0.26 (95%CI: 0.16–0.4)
Metabolic syndrome	m	X	0.38 (95%CI: 0.19–0.56)
Framingham score risk	m		0.16 (95%CI: 0.05–0.32)
Framingham relative risk index	m	X	0.47 (95%CI: 0.33–0.59)
AUDIT-C index	hb		0.08 (95%CI: 0.01–0.2)
Physical activity index (IPAQ)	hb	X	0.11 (95%CI: 0.04–0.18)
Number of medical conditions	hb		0.09 (95%CI: 0.01–0.23)
Smokers (self reported, percent)	hb		0.33 (95%CI: 0.2–0.44)
Number of regular medications	hb		0.08 (95%CI: 0–0.24)
Nutritional index	hb		0.05 (95%CI: 0.01–0.11)
Commitment to the workplace	psy		0.07 (95%CI: 0–0.18)
Quality of leadership	psy		0.07 (95%CI: 0.02–0.2)
Cognitive stress	psy		0.05 (95%CI: 0–0.16)
Emotional demands	psy		0.09 (95%CI: 0.01–0.19)
Physical demands	psy		0.32 (95%CI: 0.23–0.41)
Quantitative demands	psy		0.27 (95%CI: 0.16–0.38)
Influence at work	psy		0.31 (95%CI: 0.22–0.4)
Predictability	psy	X	0.11 (95%CI: 0.02–0.23)
Job satisfaction	psy		0.33 (95%CI: 0.23–0.47)
Possibilities for development	psy		0.39 (95%CI: 0.3–0.48)
Meaning of work	psy		0.10 (95%CI: 0.02–0.21)
Social support from colleagues	psy		0.09 (95%CI: 0.02–0.21)
Work life conflict	psy		0.05 (95%CI: 0–0.12)
Burnout	subh	X	0.14 (95%CI: 0.02–0.26)
SF-12 physical summary index	subh		0.36 (95%CI: 0.2–0.53)
SF-12 mental summary index	subh		0.04 (95%CI: 0–0.1)
Daytime sleepiness	subh		0.07 (95%CI: 0–0.17)
Work ability	subh		0.23 (95%CI: 0.09–0.37)

Legend: ^a^ an x indicates that the quadratic term was significant suggesting a possible non-linear relationship. ^b^ The explained variance in univariate analysis (R square) is presented as a measure of the effect sizes

From the multivariable models (Table 3), a model comprising work-group composition (age, gender and main type of work) explained 18% of the variation of future absence rates. Concurrent sickness absence rates in 2014 explained 49% of future absence rates in 2015. In contrast a parsimonious model comprised of psychosocial work characteristics explained 70% of the variance. Finally, a comprehensive model with eight variables (psychosocial variables and individual health variables) explained 75% of the work-group sickness absence during follow up in 2015. Table 3 provides further details for the multivariable models.

**Table 3:** Multivariable models predicting future sickness absence. The table shows the predictive accuracy (explained variance expressed as adjusted R-square) from different domains of possible predictors. Note that some factors such as main type of work or the observed sickness absence rate during the concurrent year are hardly amenable to interventions – in contrast to models containing behavioral or organizational characteristics. The confidence interval was obtained from 200 bootstrap cycles randomly selecting individuals from all participants within departments for the analytical sample

Model	Candidate predictors in model	Explained variance (adjusted R^2^)	Observed empirical confidence interval
Composition (age, gender) & context (type of work)	age, gender, main type of work (manual vs. non-manual)	0.18	(0.09–0.27)
Sick leave rate	Concurrent work-group sickness absence during the present year	0.49	(0.43–0.55)
Work ability index	work ability index, including quadratic term, adjusting for age, gender, main type of work	0.37	(0.26–0.48)
History & health behavior	smoking status, number of illnesses, number of regular medications, physical activity, AUDIT-C, self-reported sickness absence	0.49	(0.36–0.62)
Subjective health & work ability	SF12 mental and physical summary score, daytime sleepiness, exhaustion, work life conflict, work ability index	0.50	(0.35–0.64)
Work ability & Framingham index	Framingham relative risk, work ability index	0.57	(0.45–0.68)
Medical data & Framingham index	Framingham relative risk, body mass index, waist circumference, HbA1c, HDL, triglycerides, high sensitivity C-reactive protein, γ-glutamyltransferase	0.68	(0.57–0.79)
Psychosocial work characteristics	cognitive stress perception, meaning of work, commitment to the workplace, quality of leadership, predictability, possibilities for development, work life conflict	0.70	(0.60–0.81)
Parsimonious model, all domains	Framingham risk score, smoking status, meaning of work, commitment to the workplace, quality of leadership, predictability, possibilities for development, work life conflict	0.75	(0.66–0.85)

**Fig. 2:**
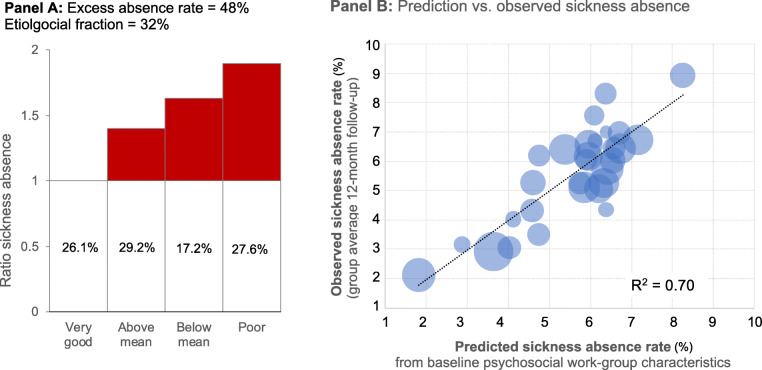
Excess sickness absence rate and etiological fraction of psychosocial work characteristics Panel **a** shows the calculation of the etiological fraction as suggested by Miettinen [19]. The vertical axis displays the ratio of sickness absence in each quartile of psychosocial work characteristics in comparison to the sickness absence in the most favorable quartile (high) as reference group. The shaded area represents the surplus attributable to less favorable psychosocial work characteristics as in the reference group. The etiological fraction is the proportion of the shaded surplus absence of the entire absence. The data is the average of 200 bootstrap cycles of weighted random sampling within each work-group. The calculation for the excess absence rate above expected baseline is as follows: (shaded area (sa)): sa = 0.29 * 0.42 + 0.17 * 0.63 + 0.28 * 0.90 = 0.48. The calculation for the etiological fraction (the amount of overall reduction if psychosocial work characteristics were like the “very good” quartile of work-groups) is: sa / (1 sa): EF = 0.48 / 1.48 = 0.32. Panel **b** displays the prediction average for the 29 work-groups from 200 boostrap cycles (horizontal axis) in comparison to the observed follow up sickness absence rate (vertical axis). The size of each bubble corresponds to the number of subjects randomly chosen from each work-group’s participants for calculation of the prediction model

The excess sickness absence attributable to more adverse psychosocial work characteristics amounted to 48% (bootstrap 95% interval: 32–67%), equivalent to an etiological fraction according to Miettinen of 32% (24–40%). The latter number represents the proportion of all sickness absence if psychosocial work characteristics in each work-group were improved to the observed benchmark of the quartile of work-groups with the most favorable psychosocial work characteristics. By comparison, the excess sickness absence attributable to all medical data and health behavior combined was estimated as 45% (29–64%), with an etiological fraction of 31% (22–38%). Figure 2 Panel a illustrates the calculation of the excess sickness rate and the etiological fraction, Panel B displays the predicted sickness absence rate from psychosocial variables measured at baseline for all 29 work-groups vs. the observed sickness absence rate at follow-up.

## Discussion

We conducted a prospective multi-site cohort study in a large German automotive production group. We found that work-group averaged perceived psychosocial work characteristics explained 70% of the variance of in work-group sickness absence rates 12 months later. We showed that the excess sickness absence rate in the three quartiles with work-groups of less favorable psychosocial work characteristics amounted to 48%. In other words, if all psychosocial work characteristics could be improved to the level of the best quartile, our data predict an overall reduction in sickness absence rate by 32%. For comparison, hypothetically reducing all health behavior and medical risk factors to the “unavoidable” level observed in the best quartile of work-groups would predict a reduction of sickness absence rate by 31%.

To our knowledge, this is one of the few studies examining data at the work-group rather than the individual level [17, 18]. Our study arrives at similar estimates as did the Danish registry studies obtained from individual person follow-up data, which more than a decade ago estimated the etiological fraction of sickness absence rates attributable to seven psychosocial work characteristics at 29% [11]. Work conducted in the past century following up individual subjects (i.e. the Whitehall II study [37]) strongly suggested a possible link between adverse psychosocial work characteristics and sickness absence [38–40]. Many companies now use employee assistance programs to support individuals with mental challenges. The aim is to improve personal coping and resilience skills, regardless of whether the origin of the stress arises from private matters or work related issues.

However, health and safety regulations strictly mandate that technical or organizational measures have priority over individual protection. This holds for example for exposure to radiation, chemical hazards, noise, heat or cold. No one would aim to reduce exposure to toxic chemicals by offering individual personal training if technical or organizational measures were available or simply recruit employees deemed more biological resilient to the toxin. However, exposure to unfavorable psychosocial work characteristics at the work-group level often does not experience the same rigorous approach. Using the work-group averaged perceived psychosocial work characteristics from random subsampling of the all participants as the best possible proxy estimate of the population exposure, we corroborate previous findings at the ecological level [17]. We add the perspective of simultaneously assessing health behavior and objective medical data at the individual level [33, 41]. Because we chose the quartile or real existing work-groups with the best psychosocial work characteristics as the reference, the excess sickness absence rate potentially ameliorable reflects a realistic target for organizational measures. Further, the diversity of identified variables points to differing intervention strategies similar to the array of health promotion efforts targeting risks e.g. from smoking over nutritional behavior to physical inactivity.

What are the biological or behavioral pathways possibly explaining this observation? More than half of medically certified sickness absence in Germany is accounted for by three groups of conditions: flu-like respiratory tract infections, musculoskeletal disorders and mental illness [9]. For each of these groups of conditions, psycho-neuro-immunological research has shown increased disease propensity under chronic stress or adverse psychosocial conditions. For example, the innate immune system as well as the specific immune system (naïve T-helper cells) exhibit lower functioning in stressed individuals, increasing the probability of symptomatic viral infections [42]. Likewise, the probability of recurrent back-pain episodes or the manifestation of mental illness is higher under adverse psychosocial environments. Further, flu like illness, back-pain, depression, anxiety, exhaustion or burnout occur along a disease continuum from almost absent symptoms to severe handicap and inability to work. We posit that adverse psychosocial work-conditions shift the individual threshold at which one deems herself unable to continue working [i.e. obtaining a medical certification for sickness absence) towards absence. In contrast, improved psychosocial work characteristics may lift the sickness threshold and support remaining non-sick. Earlier prospective studies indeed showed that improved quality of leadership reduced long-term sickness absence in employees with moderate depressive symptoms [43].

What are the clinical implications of our findings? Up until now, the workplace wellness movement has almost exclusively focused on health risk assessments that target avoidable adverse health behaviors or medical risk factors, particularly for cardiovascular disease [44–46]. Rigorous scrutiny of such programs revealed little effect on organizational measures or on health service usage patterns [8]. The eight identified psychosocial predictors, namely cognitive stress perception, meaning of work, commitment to the workplace, quality of leadership, predictability, possibilities for development or work life conflict point to options for organizational interventions at the work-group level.

Several caveats of our study require consideration. First, the data were obtained from a predominantly white, male work-force and may not be generalized to female or non-white employees. Second, the national context from which our data were obtained (the German social security system) may differ from others in which the individual may encounter greater financial disincentive associated with sickness absence. Thus, the German context may be biased towards higher sickness absence rates than those observed in other countries. In the present context this turns into a strength of the study, as absence or presence on a specific working day is a binominal outcome (absenteeism), much easier to objectify that being present with health complaints or impaired workability (presenteeism).

A further caveat is the possible selection bias arising at the company level when choosing work-groups for participation. The core question is, whether leaving the selection of candidate work-groups to the human resource department may have introduced a selection bias affecting our results. For example, specifically selecting departments on both ends of the distribution for favorable and adverse psychosocial work characteristics might have induced a type of case control study, where hidden confounders account for the difference in absence rates and not the relationships elucidated in this investigation. While we cannot fully rule out this possibility, we had the opportunity to follow the company for further four years with different work-groups. During the later years, no objective absence data was available to us. However, cross-sectional correlation analysis between self-reported absence, work-ability and psychosocial work characteristics do not hint to any major deviation from our findings. Further, the observed sickness absence rate of 4.6–5.1% per year in the selected work-groups was comparable to sickness absence rates reported for industrial employees from data collected by the German statutory health insurance plans for the same period (4.7%) [9]. In this context it is noteworthy that we observed a 70% larger objectively recorded sickness absence rate as obtained from the company records for the entire work-group as compared to the rate calculated from self-reporting amongst participants. This difference was substantially larger than that reported when comparing individual self-report with individual social security data in the Whitehall II study [34]. We interpreted this as a potential selection and recall bias. Thus, in contrast to the recommendation by the Whitehall researchers [34], we considered substituting objective recorded sickness absence by self-reported data for applying a typical multilevel approach inappropriate.

A further limitation is that across the seven sites and different branches of the organization, the level of differentiation that was made available to us differed. Thus, the largest work-group with several hundred participants in reality broke down along further hierarchies into several sub-entities that would have allowed further differentiation. While we knew about this further differentiation and even had psychosocial work characteristics data supporting considerable heterogeneity within the larger unit, we were unable to obtain the outcome of objective absence data at finer granularity from the company.

The strength of this study is that it extends research from the last century following up state employees on the individual level (i.e. Whitehall II) to data from a highly competitive industrial production context in the second decade of the twenty-first century. As we analyzed the data at the relevant ecological work-group level, we were able to simultaneously consider contextual factors (i.e. psychosocial work characteristics) and individual health factors at the person level.

## Conclusion

Our study showed that a large fraction of future sickness absence rates at the work-group level can be explained by psychosocial work characteristics. We were able to predict future sickness absence rates from work-group averaged perceived psychosocial work characteristics with surprising accuracy. Our models predict that removing adverse psychosocial work characteristics at the work-group level by means of organizational measures to the realistic level of the quartile with the most favorable ratings would have a similar effect on sickness absence rates as eliminating risky heath behaviors and medical problems to the level found in the healthiest quartile work-groups. Health and safety rules and regulations developed to ameliorate physical hazards mandate that action at the organizational level towards improving psychosocial work characteristics is a must and not a desirable option.

## Supplementary Information

**Additional file 1.**

## Data Availability

The datasets generated and/or analyzed during the current study are not publicly available due regulation by the IRB with respect to the GDPR consent given by participants original consent form. However, an anonymized data-set with randomly altered values maintaining the original relationships is available from the corresponding author on reasonable request.
